# ﻿An update on the taxonomy of *Calamagrostisnagarum* (Bor) G.Singh and its allies (Poaceae, Agrostidinae): morphometrics and micro-morphology

**DOI:** 10.3897/phytokeys.212.89253

**Published:** 2022-11-04

**Authors:** Dileshwar Prasad, Ravindra Kumar, Shubham Jaiswal, Rekha Yadav, Smita Tiwari, Priyanka Agnihotri

**Affiliations:** 1 Plant Diversity, Systematics & Herbarium Division, CSIR–National Botanical Research Institute, Rana Pratap Marg, Lucknow–226001, India CSIR–National Botanical Research Institute Lucknow India; 2 Academy of Scientific and Innovative Research (AcSIR), Sector 19, Kamla Nehru Nagar, Ghaziabad–201002, India Academy of Scientific and Innovative Research Ghaziabad India

**Keywords:** Cool season grass, *Deyeuxia*, lectotypification, micro-morphology, taxonomy, Western Himalaya

## Abstract

*Calamagrostisnagarum*, previously considered to be a poorly known species, has been reassessed taxonomically. It is a member of *C.lahulensis*-*C.scabrescens* complex and may be segregated by morphological characters such as the presence of pilose hairs on adaxial surface of leaf blades, spreading panicle branches, filiform awn and nerve prolongation of lemma. Besides, the micromorphology of adaxial surface of leaf blades, dorsal surface of glume and lemma differentiates *Calamagrostisnagarum* from its allies, *C.lahulensis* and *C.scabrescens*. It is known from Nagaland and Uttarakhand, India, and Bhutan. In this study, we have provided an emended description of the species, a discussion of its habitat and distribution, and taxonomic notes along with field photographs and photo plates for its correct identification. In addition, we also lectotypify the names *C.lahulensis* and *C.scabrescens*.

## ﻿Introduction

The genus *Calamagrostis* Adans. s. str., including *Deyeuxia* Clarion ex P.Beauv., belongs to the subtribe Agrostidinae (Poaceae, Pooideae, Poeae) and includes about 130 species globally (Soreng et al. 2022). Several American species previously recognized under *Calamagrostis* have been transferred to the genera *Cinnagrostis* Griseb., *Greeneochloa* P.M. Peterson, Soreng, Romasch. & Barberá, *Laegaardia* P.M. Peterson, Soreng, Romasch. & Barberá, *Paramochloa* P.M. Peterson, Soreng, Romasch. & Barberá, *Peyritschia* E. Fourn, and *Deschampsia* (Saarela et al. 2017; Peterson et al. 2019). *Calamagrostis* s. str. is characterized by plants rhizomatous or not, with or without extravaginal branching; lemma awns straight or slightly bent, readily distinguished from callus hairs, inserted at base to middle or rarely near the apex; rachilla extension penicillate hairy along the length or glabrous, or, if rudimentary, then callus with long hairs; callus hairs 1/10‒3/4 as long as the lemma in length or longer than lemma; lodicules entire and lanceolate, sometimes with an isolated lateral lobe, glabrous; ovary glabrous, distinctly sulcate, hilum 1/6‒1/3 the grain in length (Peterson et al. 2019). It is also similar to the genus *Agrostis* L. and both genera have several notoriously difficult species complexes, as well as hybrids at the global level (Howard et al. 2009; Paszko and Nobis 2010; Paszko and Ma 2011; Paszko 2012b, 2013). In India, *Calamagrostis* s. str. comprises about 22 species which are mainly confined to Himalayan region (Bor 1960; Paszko 2012a; Paszko and Soreng 2013; Kellogg et al. 2020; Prasad et al. 2021) and formed several species complexes such as the *C.emodensis* Griseb. complex, the *C.epigeios* (L.) Roth complex, the *C.lahulensis* G.Singh-*C.scabrescens* Griseb. complex, and the *C.pseudophragmites* (Haller f.) Koeler complex, however, the majority of these have been recently resolved (Paszko and Ma 2011; Paszko 2012a, 2012b, 2013, 2014a, 2014b). *C.lahulensis*-*C.scabrescens* complex is characterized by the presence of rachilla with penicillate hairs which is as long as or more than the length of lemma, callus hairs shorter than half of the length of lemma and awn inserted 2/3^rd^ from base to near the tip on the dorsal side of lemma (Bor 1960; [Bibr B20][Bibr B22], 2016).

*Calamagrostisnagarum* (Bor) G.Singh was originally described by Bor (1938) under the genus *Deyeuxia* Clarion ex P. Beauv as *D.nagarum* Bor. At the time of its description, it was only known from the type locality Naga Hills, Nagaland, India, but was later also reported in the grass flora of Bhutan (Noltie 2000). Furthermore, it has been documented in the updated checklist of grasses of Uttarakhand, India (Kandwal and Gupta 2009). The diagnosis and keys, provided by Bor (1940, 1960), Shukla (1996) and Noltie (2000), are overlapping with *C.elatior*, *C.lahulensis* and *C.scabrescens*, therefore, do not adequately segregate *C.nagarum* from them. Although the morphological descriptions provided by Bor (1940) and Shukla (1996) exactly correspond to *C.nagarum*, the features of panicles, narrow and up to 07 cm long, provided by Noltie (2000), have created confusion for the species identity. Furthermore, Bor (1940) and Shukla (1996) stated that *C.nagarum* is morphologically similar to *C.lahulensis* G.Singh and *C.scabrescens* Griseb., whereas Noltie (2000) assumed that it is more similar to *C.elatior*, perhaps on the basis of pilose hairs on adaxial side of leaf blades. Since the existing taxonomic literature is unable to distinguish *C.nagarum* from its allied species, we conducted a taxonomic reassessment of *C.nagarum* to clarify its identity.

In the present study, we also lectotypify the names *C.lahulensis* G. Singh and *C.scabrescens* Griseb. since no specimen was selected as type specimen for either name (Art. 9.3 of ICN; Turland et al. 2018). Both species were originally described by Grisebach, a German botanist, who worked in Kew herbarium and studied Hooker’s specimens of grasses collected from India and described and/or recorded a total of about 43 taxa within the Agrostideen group including *Calamagrostispulchella* Griseb. (replaced synonym of *C.lahulensis*) and *C.scabrescens* Griseb.

## ﻿Materials and methods

This study is based on an examination of herbarium specimens as well as field collections belonging to *Calamagrostislahulensis*, *C.nagarum* and *C.scabrescens*. Self-collected specimens, deposited at CSIR-National Botanical Research Institute, Lucknow (**LWG**) herbarium were gathered during botanical trips in several localities of Western and Eastern Himalaya. The following Indian Herbaria: Botanical Survey of India, Regional Centre, Dehradun (**BSD**), Central National Herbarium, Botanical Survey of India, Howrah (**CAL**), Indian Council of Forestry Research and Education, Dehradun (**DD**), University of Kashmir, Srinagar (**KASH**) and LWG were also consulted in person (see Appendix 1). The identification of *Calamagrostis* spp. was done through a consultation of international, national and regional floras and taxonomic studies (Hooker 1896; Bor 1938, 1940, 1960; Chowdhary and Wadhwa 1984; Aswal and Mehrotra 1994; Shukla 1996; Gaur 1999; Noltie 2000; Lu and Phillips 2006; Pusalkar and Singh 2012; Paszko et al. 2013; Paszko 2014a; Paszko et al. 2017; Prasad et al. 2021). Besides, specimens that belong to *C.lahulensis*, *C.nagarum* and *C.scabrescens* were identified by their respective protologue (Grisebach 1868; Bor 1938). Morphological measurements were recorded from 26 spikelets of *C.lahulensis*, 23 spikelets of *C.nagarum* and 42 spikelets of *C.scabrescens*, from the specimens housed at LWG, usually on one spikelet from one individual, using a Stereo Zoom Trinnocular microscope equipped with a MC 120 HD camera. Photographs of *C.nagarum* were also taken. The recorded quantitative data of morphological characters (Table 1) of *C.lahulensis*, *C.nagarum* and *C.scabrescens* were subjected to univariate variance analysis. For Principal component analysis (PCA), the statistic software XLSTAT BASIC + (https://www.xlstat.com/en/) was used. The morphological data recorded from spikelets (except panicle length and ligule length) were included in the PCA analysis. To analyse the morphological boundaries between *C.lahulensis*, *C.scabrescens* and *C.nagarum*, a scatter plot of PCA loadings ≥ 0.70 of the selected morphological characters was conducted. We prepared distribution map of *C.nagarum* by using DIVA-GIS computational program (Hijmans et al. 2001) based on examined specimens and localities documented in Bor (1960) and Noltie (2000).

**Table 1.** Morphological characters used in the present study.

Morphological characters	Character abbreviation (unit)
Panicle length	PNL (cm)
Ligule length	LIGL (mm)
Lower glume length	LGL (mm)
Lower glume width	LGW (mm)
Upper glume length	UGL (mm)
Upper glume width	UGW (mm)
Lemma length	LL (mm)
Palea length	PL (mm)
Awn length	AL (mm)
Rachilla hairs length	RHL (mm)
Lemma nerves prolongation length (intermediate nerve and lateral nerve)	LNP (mm)
Lemma base to awn insertion point length	LBTAIP (mm)
Awn insertion point to lemma tip length	AIPTLT (mm)
Ratio: lower glume length to upper glume length	LGL/UGL
Ratio: lower glume width to lower glume length	LGW/LGL
Ratio: lemma length to lower glume length	LL/LGL
Ratio: palea length to lemma length	PL/LL
Ratio: rachilla hair length to lemma length	RHL/LL
Ratio: awn length to lemma length	Al/LL
Ratio: awn insertion points to lemma tip, to lemma base to awn insertion point	AIPTLT/LBTAIP

For the micro-morphological study of *C.lahulensis*, *C.nagarum*, and *C.scabrescens*, we examined leaf blades, glumes, and lemmas from the collections *D. Prasad et al. 326642, 339372* and *326717* (LWG), respectively. All the materials were fixed in formalin-acetic-alcohol (ratio 1:3:1) solution for 48 hr. The samples were then dehydrated with increasing strengths of ethyl alcohol solutions. Thereafter, the prepared samples were examined using a FEI QUANTA250F scanning electron microscope (SEM) in low vacuum mode (Doğan 1988) at CSIR-National Botanical Research Institute, Lucknow, India.

Specimens of *C.lahulensis* and *C.scabrescens* matching the criteria of original material were examined online at Montpellier University, Montpellier, France (MPU), National Museum of Natural History, Paris, France (P), Natural History Museum, Vienna, Austria (W), Royal Botanic Garden, U.K. Scotland, Edinburgh (E), Royal Botanic Gardens Kew, U.K. England, Kew (K) and The Natural History Museum, London (BM), as well as in person at CAL.

## ﻿Result and discussion

### ﻿Morphological variation and morphometrics

**Figure 1.**
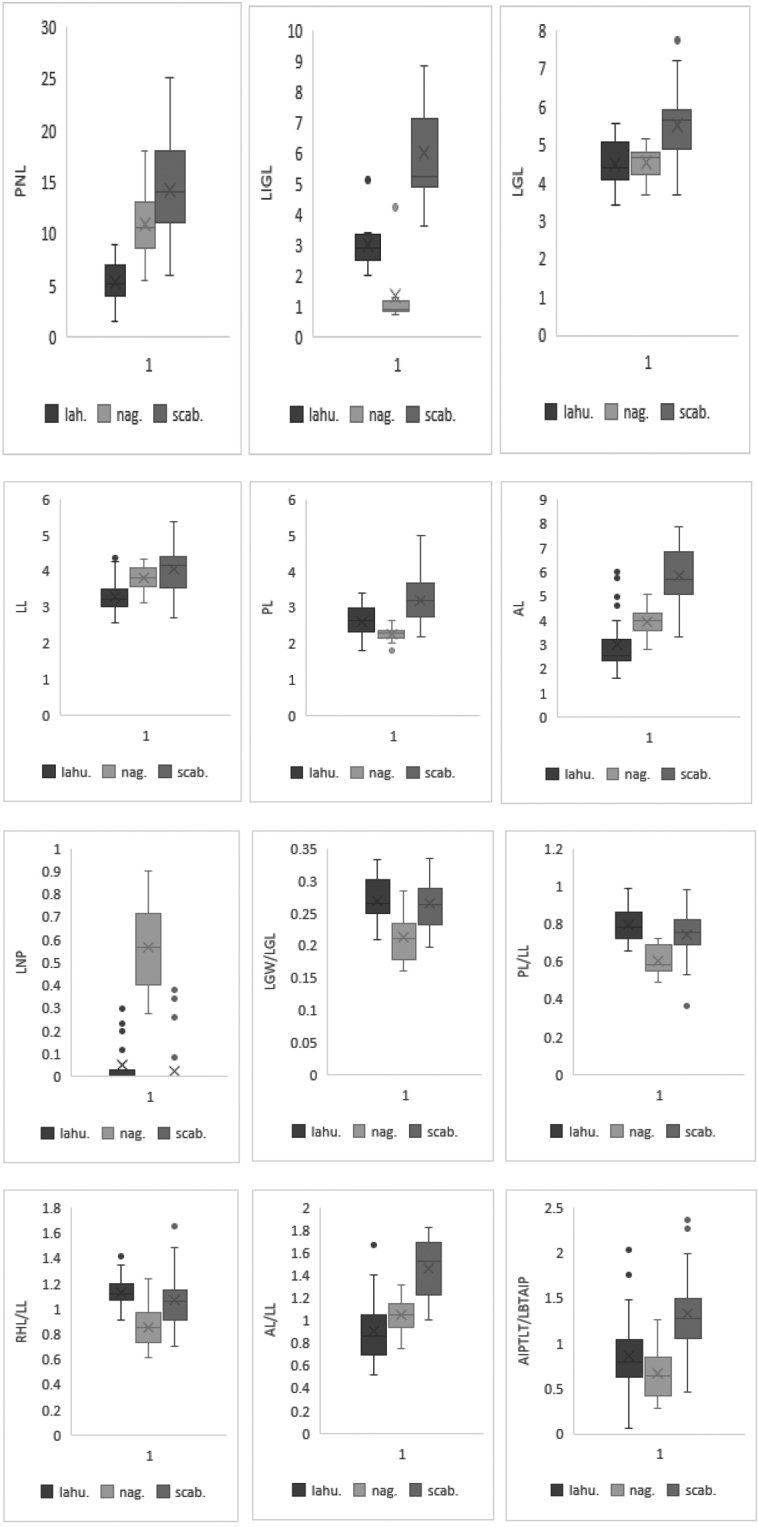
Box plots for selected morphological variables showing mean (cross), mean ± SD (box), outliers (dot) and range of variation (whiskers) for *C.lahulensis* (lah.), *C.nagarum* (nag.) and *C.scabrescens* (scab.).

Analysis of selected morphological characters of *Calamagrostisnagarum* revealed that most of the characters overlap with *C.lahulensis* and *C.scabrescens* except nerve prolongation of lemma (LNP) and ratio of lower glume width to lower glume length (LGW/LGL) (Fig. 1). The lemma apex in *C.lahulensis* and *C.scabresens* is erose (like four small unequal lobes), usually without nerve prolongation or rarely with a nerve prolongation, which is 0.08–0.26 (0.38) mm long, whereas in *C.nagarum* the nerve prolongation of lemma is (0.3–)0.4–0.7(–0.9) mm long. We have observed a nerve prolongation of lemma only in six spikelets of *C.lahulensis*, four spikelets of *C.scabrescens* and all spikelets of *C.nagarum*. A strongly geniculate and long awn is common in *C.scabrescens*, but straight and short awns are also observed in some spikelets in which the glume margin is ciliate. The point of awn insertion on the dorsal side of the lemma is highly variable in *C.lahulensis*, inserted on lower 2/3^rd^ to the tip of the lemma, and similar variation is also observed in *C.nagarum*. In *C.scabrescens*, however, the awn is inserted on the middle to lower 2/3^rd^ of lemma. The ratio of palea to lemma is constant in a range of (0.65–)0.70–0.85(–0.99) in both *C.lahulensis* and *C.scabrescens* while a narrower range of variation, (0.48–)0.55–0.68(–0.75) is observed in *C.nagarum*. The ratio of rachilla hair length to lemma length in *C.lahulensis* ranges from (0.90–)1.06–1.19(–1.35) and has a wide range of variation in both *C.nagarum* and *C.scabrescens*.

**Figure 2.**
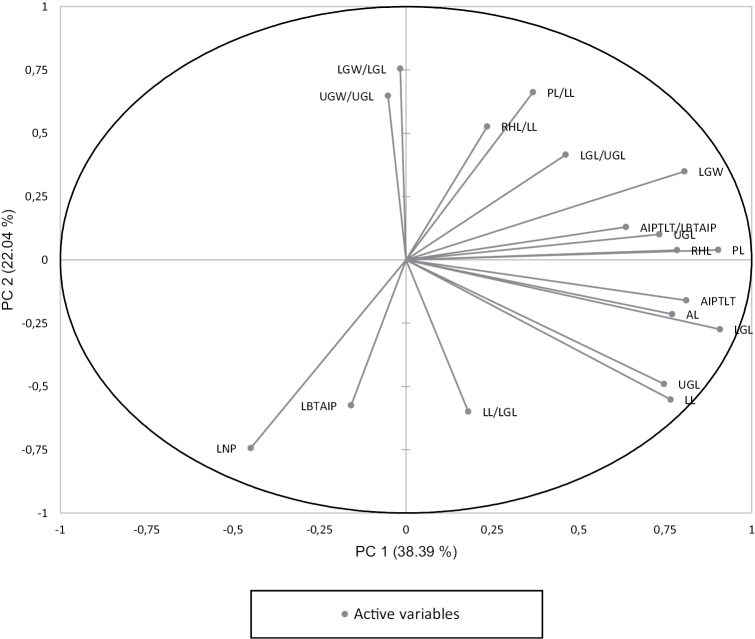
Projection of variables on principal component (PC1 × PC2) scored for *C.lahulensis*, *C.nagarum* and *C.scabrescens*.

**Figure 3.**
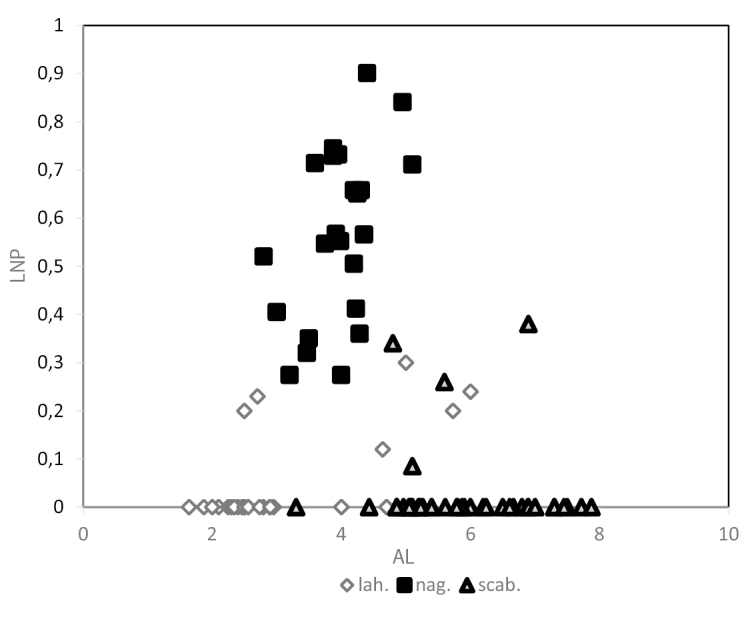
Scatterplot of lemma nerve prolongation length (LNP) again awn length (AL) for *C.lahulensis*, *C.nagarum* and *C.scabrescens*.

The first principal component (PC1) had relatively high (positive or negative) loading for lower and upper glume length and width, lemma length, rachilla hairs length, awn length, awn insertion points to lemma tip length and palea length, however, PC2 had relatively high loading for lemma nerve prolongation and ratio of lower glume width to lower glume length (Fig. 2). According to PC1 vs. PC2, *C.nagarum* is separable from *C.lahulensis* and *C.scabrescens* on the basis of nerve prolongation of lemma (Fig. 3), whereas *C.lahulensis* and *C.scabrescens* overlap.

### ﻿Micro-morphology of leaf blades, glume, and lemma

**Figure 4.**
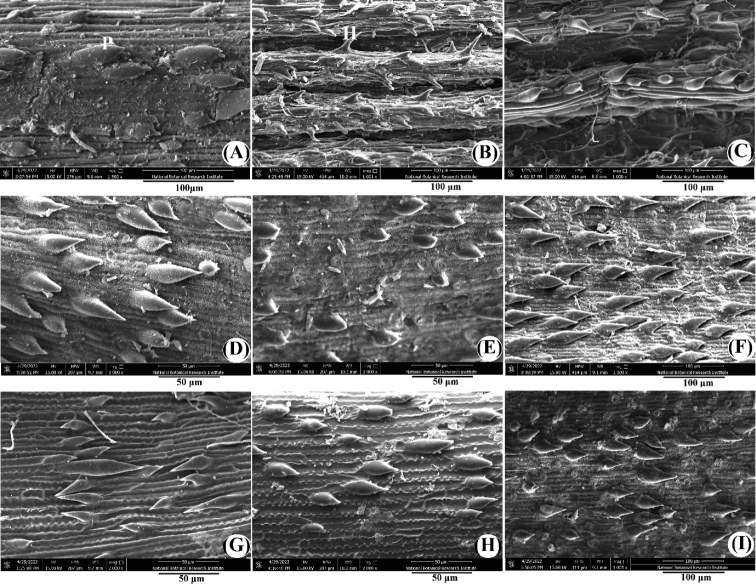
SEM morphology of adaxial side of leaf blades (**A–C**), dorsal surface of glume (**D–F**) and dorsal surface of lemma (**G–I**) in *C.lahulensis* (**A, D, G**), *C.nagarum* (**B, E, H**) and *C.scabrescens* (**C, F, I**).

**Figure 5.**
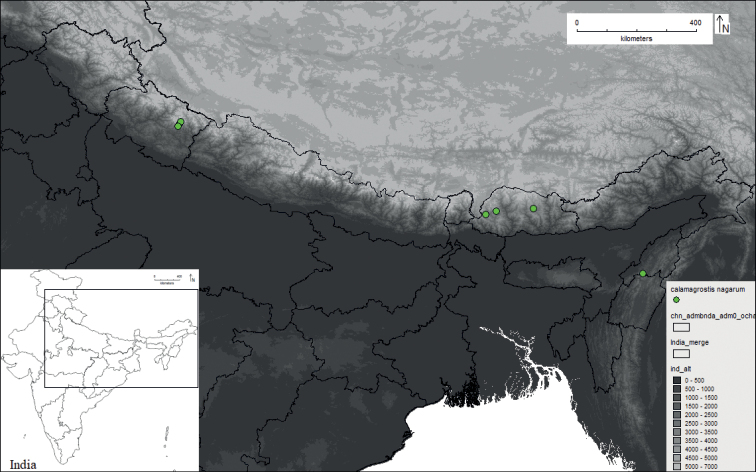
Geographic distribution of *Calamagrostisnagarum*.

On the adaxial surface of leaves, *C.lahulensis* has less prominent grooves or sometimes absent (Fig. 5A), while in *C.nagarum* (Fig. 5B) and *C.scabrescens* (Fig. 5C) grooves are prominent with deep furrows. Rigid spicules of adaxial side of leaf blades are of two different types: prickle (short-pointed apex) and hooked (long pointed apex). The former is usually densely or sometimes sparsely present in *C.lahulensis* (Fig. 4A), while in *C.nagarum* (Fig. 4B) and *C.scabrescens* (Fig. 4C) both are present. In *C.nagarum*, spicules are arranged in four rows along the grooves (Fig. 4B), while in *C.scabrescens* there are two rows of densely and sparsely arranged spicules on grooves (Fig. 4C). Length of spicules is 49.7–57.2 µm long (Fig. 4A) and 42.5–54.9 µm long (Fig. 4C) in *C.lahulensis* and *C.scabrescens*, respectively, but in *C.nagarum* length of spicules is 21.7–45.60 µm long (Fig. 4B). However, the pilose hairs, present on the adaxial surface of leaf blades in *C.nagarum*, shed during the preparation of the sample. The dorsal glume surface is scabrous because of rigid spicules in all three species (Fig. 4D–F). Prickles on dorsal glume surface are sparsely arranged in *C.nagarum* (Fig. 4E), while in *C.lahulensis* (Fig. 4D) and *C.scabrescens* (Fig. 4F) prickles are absent, but hooks are densely arranged. *C.lahulensis* and *C.scabrescens* have spicules 35.8–47.3 µm long (Fig. 4D) and 47.27–75.01 µm long (Fig. 4F), respectively, that are comparatively longer than *C.nagarum*, in which spicules are usually 19.51–26.43 µm long (Fig. 4E). The dorsal lemma surface shows a high degree of variability among all three species (Fig. 4G–I). The hooks are arranged antrorsely and retrorsely in *C.lahulensis* and are of two different lengths, short (27.42–37.80 µm long) and long (0.65–0.75 µm long) (Fig. 5G). However, in both *C.nagarum* and *C.scabrescens* prickles are 25.82–63.38 µm long (Fig. 4H) and 25.37–39.51 µm long (Fig. 4I), respectively.

### ﻿Taxonomic treatment

#### ﻿ Calamagrostis nagarum

Taxon classificationPlantaePoalesPoaceae

(Bor) G.Singh in Taxon 33(1): 94 (1984); Shukla, Grass. N. E. India, 47 (1996)

476B1935-4493-5091-B71A-D54F8355D4B9

Deyeuxia nagarum Bor in Indian Forest Rec., Bot. 1: 69 (1938); Bor, Fl. Assam, 5: 145 (1940); Bor, Grass. Burma, Ceylon, India & Pakistan, 399 (1960). Basionym.

##### Type.

India. Nagaland [earlier in Assam], Naga hills, Japvo range, 9,500 ft [2895 m], September 1937, *NL Bor 2834* (holo. K: K000032378, digital image!).

##### Amended description.

A perennial, rhizomatous, robust grass, 50–100 cm tall. Culms 40–80 cm long, simple, terete, glabrous, 2–3 nodes below the panicle. Node glabrous, compressed. Leaf sheaths split, overlapping, loose, connate toward base, smooth, sometimes scaberulous. Leaf blades 7–20 × 0.3–0.7 cm, flat, narrowly-linear, adaxial scabrous with distantly pilose and abaxial surface scabrous; apex attenuate; margin scabrid. Ligules 0.75–4.2 mm long, membranous, adaxial surface glabrous, abaxial surface scabrous; apex obtuse, lacerate. Inflorescence a panicle, 5–18 × 5–8 cm, very lax with spreading branches; lower panicle branches paired or in whorls of 3–5; 1–8 cm long, almost smooth or sometime scabrous, filiform, flexuous. Rachis slender, glabrous or scabrous. Spikelets 5.1–6.7 × 1.5–2 mm, lanceolate to wedge shaped at maturity, bearing 1-floret, disarticulating above the glume and below the floret, greenish with pink tinged; glumes subequal, persistent; floret hermaphroditic. Pedicel shorter than spikelet, slender, scabrous. Lower glume 3.7–5.2 × 0.65–1.3 mm, 1-nerved, 1-keeled, lanceolate, greenish with pink tinge near margin, scaberulous to somewhat glabrous; apex acuminate; margin narrowly hyaline, entire; keel scabrous. Upper glume 3.8–5.4 × 0.87–1.34 mm, 3-nerved, 1-keeled, lanceolate, greenish with pink tinged, scabrous; apex acuminate; margin narrowly hyaline, entire; keel scabrous. Callus evenly bearded, hairs 1.1–1.9 mm long, nearly half of the length of lemma or shorter. Lemma 3.1–4.3 × 1.0–1.6 mm, 5-nerved, membranous, surface scaberulous with papillate, awned; apex acute with 4-nerve prolongation 0.27–0.9 mm long; margin hyaline. Rachilla 1.2–1.9 mm long, penicillate hairy, usually bare at base; rachilla with hairs 2.5–4.9 mm long. Awn 2.8–5.1 mm long, straight, filiform, slender, scabrous-antrorse, exerted from the spikelet, arising from above the middle of lemma back. Palea 1.8–2.7 mm long, 2-nerved, 2-keeled, hyaline-membranous, rounded on back; apex slightly bifid. Lodicules 2, 0.7–0.8 mm long, lanceolate. Stamens 3; anthers 1.5–2.3 mm long, narrowly linear. Mature caryopsis not seen.

##### Phenology.

September to October (flowering and fruiting).

##### Habitat and distribution.

**Figure 6.**
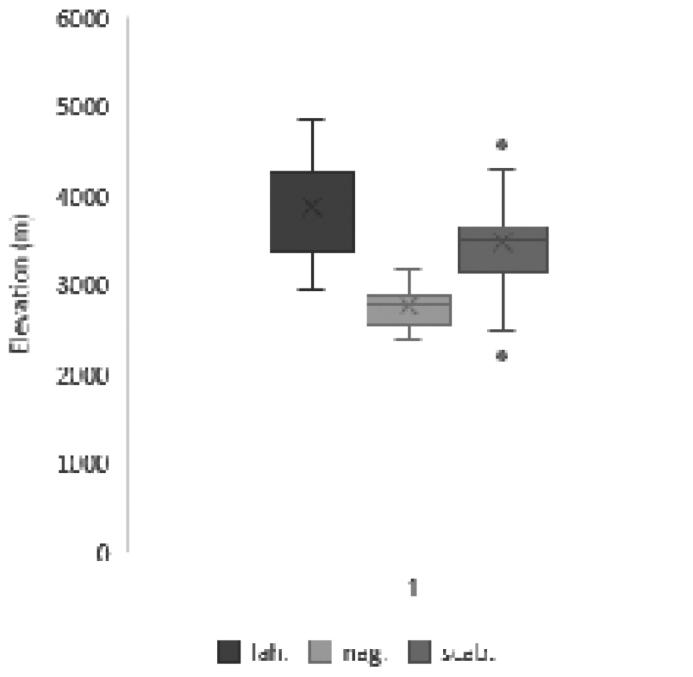
Box plot for vertical distribution of *C.lahulensis*, *C.nagarum* and *C.scabrescens*.

*Calmagrostisnagarum* was discovered in the Japvo range of Naga Hills situated in Nagaland, which is geographically located in the eastern region of Assam, southernmost of Arunachal Pradesh and northern Manipur, India and close to the political boundary of Myanmar. Approximately 3% of the total geographical region of Nagaland is part of the Himalayan region, while the rest of the region is situated in a complex mountain system forming Naga Hills. Previously, it was only known from the type locality in a sub-temperate region at about 2800 m elevation and was considered to be endemic for that geographic range (Bor 1938, 1940, 1960; Shukla 1996). Later, it was recorded from Bhutan, geographically located in Eastern Himalayas, by Noltie (2000), where it was found not only on damp shady cliffs in blue pine and oak forest but also in riverbanks and scrubland, at 2400–2840 m elevation. Recently, it was documented in an updated checklist of grasses of Uttarakhand, Western Himalaya (Kandwal and Gupta 2009), but this geographic range was not included by Kellogg et al. (2020) as part of the species distributional range. During the present study, we collected specimens of *C.nagarum* from Pindari Valley, located in Bageshwar district of Uttarakhand, and confirmed its occurrence in Western Himalaya (Fig. 5). It was found growing in *Danthonia* grassland in association with *Polygonum* sp., *Anagalis* sp., and *Gaultheria* sp., at 3000–3050 m elevation in Phurkia village and, in Dhakuri top at about 2900 m elevation on forest margin as well as on Pindar riverbank in Dwali village at 2500–2800 m elevation. The vertical distribution shows *C.nagarum* is mainly confined to 2500–3100 m elevation, below the tree line, at about 3,300 m elevation, whereas *C.lahulensis* is widely distributed above the treeline at about 3350–4200 m elevation, which overlaps with the elevation range of *C.scabrescens* (Fig. 6).

##### Taxonomic notes and allied species.

**Figure 7.**
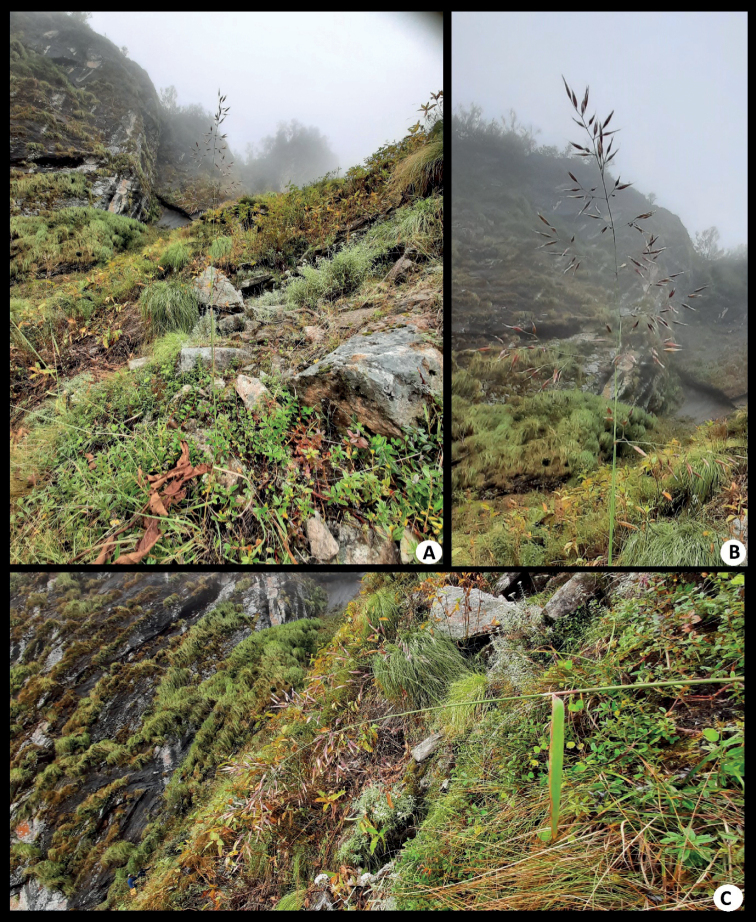
*Calamagrostisnagarum*: **A** habit **B** panicle **C** leaf blade and panicle. [Photos were taken through Samsung F41 by the first author, correspond to D. Prasad et al. 339372].

**Figure 8.**
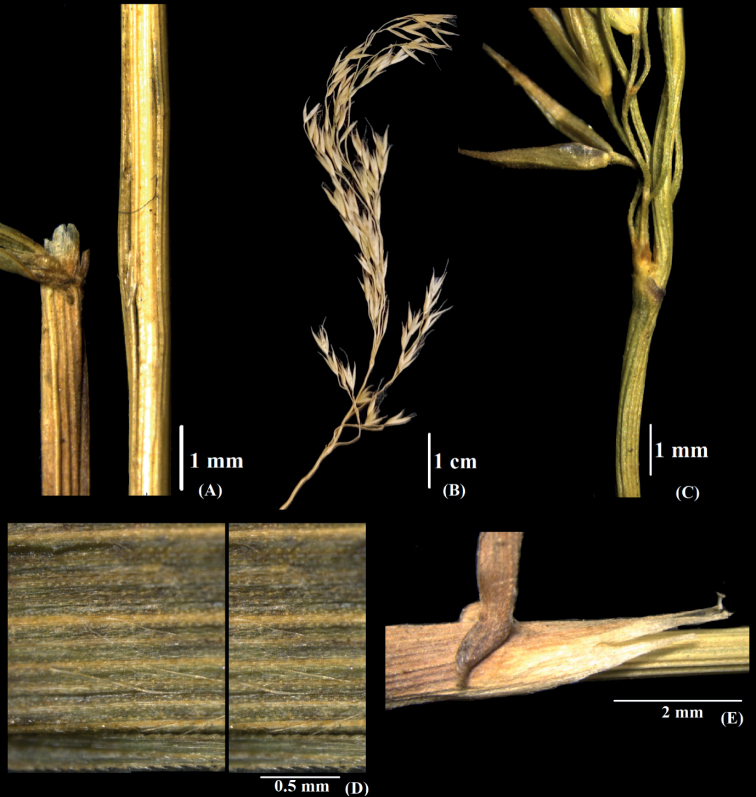
*Calamagrostisnagarum*: **A** culm and ligule **B** Panicle **C** lower panicle branch **D** leaf blade, adaxial view **E** ligules. [Photographs: **A–C** from Prasad et al. 339324 and **D–E** from D. Prasad et al. 339372].

**Figure 9.**
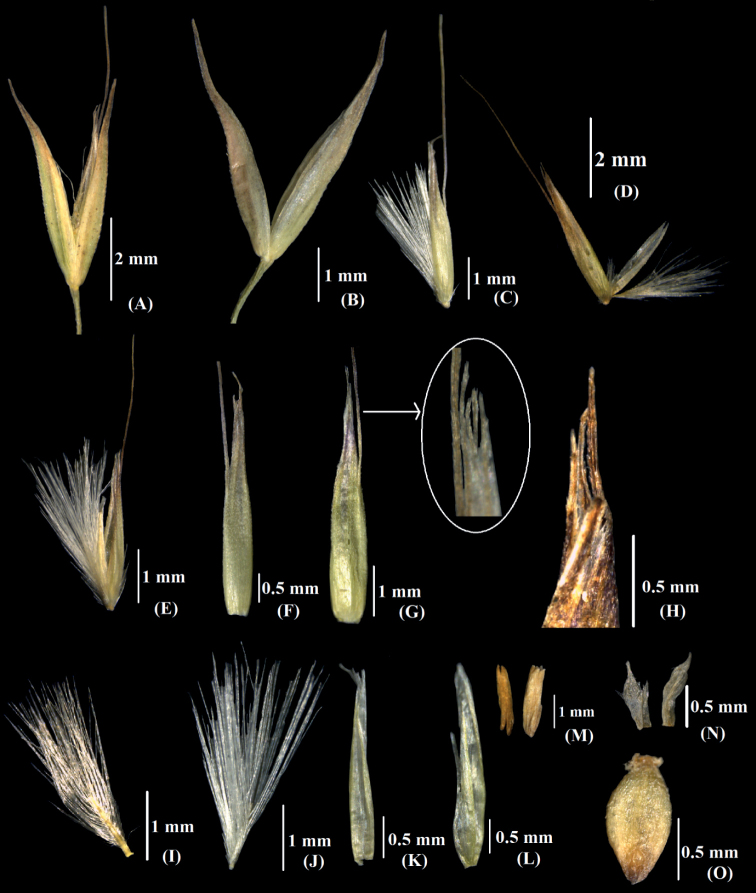
*Calamagrostisnagarum*: **A** spikelet **B** remove glumes **C** floret **D, E** Open floret **F, G** lemma, **H** lemma apex **I, J** rachilla, **K, L** palea **M** anthers **N** lodicules **O** caryopsis. [Photographs: **A–E**, **H–I** and **L–O** from D. Prasad et al. 339324 and **F–G** and **J–K** from D. Prasad et al. 339372].

*Calamagrostisnagarum* should be placed in *C.lahulensis*-*C.scabrescens* complex because of its rachilla with penicillate hairs equal to longer than lemma, callus hairs shorter than half of the lemma and awn inserted at about middle to tip of the lemma. Within this complex, it should be recognized by the presence of pilose hairs on adaxial surface of leaf blade, widely spreading panicle branches, nerve prolongation of lemma (0.3–)0.4–0.7(–0.9) mm long and filiform awn within this complex (Figs 7, 8, 9). *Calamagrostisnagarum* is more similar to *C.lahulensis* than to *C.scabrescens*. It differs from *C.lahulensis* in panicle length [(5–)8.1–12(–18) cm long vs. (1.5–)4–7(–9) cm long], lower branches of panicle [spreading with (1.0–)3–7(–8) cm long vs. ascending with (0.5–)1–3(–3.5) cm long] and, from the latter in culm (glabrous vs. scabrous), ligule length [(0.75–)0.83–1.2(–1.36) mm long vs. (3.6–)4.9–7.1(–8.84) mm long], lower glume width [(0.65–)0.81–1.1(–1.3) mm long vs. (1.1–) 1.3–1.5 (–1.7) mm long)] and awn length [(1.6–)2.3–3.2(–5.7) mm long vs. (3.3–)5.1–6.8(–7.9) mm long]. *Calamagrostisnagarum* is somewhat similar to another member of this complex, *C.nandadeviensis*, in having (5–)8.1–12(–17) mm long panicles, but differs from the latter in culm pubescence (glabrous vs. scabrous), ligule length [(0.75–)0.83–1.2(–1.36) mm long vs. (6.1–)6.5–7.5(–8.1) mm long], upper glume nervation (3-nerved vs. 1-nerved) and ratio of palea length to lemma length [(0.49–)0.54–0.62(–0.72) vs. (0.75–)0.77–0.85(–0.90)]. Along with this, *C.himalaica* (L.Liu ex Wen L.Chen) Paszko, reported from China and Myanmar, and *C.nyingchiensis* (P.C.Kuo & S.L.Lu) Paszko, restricted to China (Paszko 2015; Paszko 2016) are also members of the *C.lahulensis*-*C.scabrescens* complex. *C.nagarum* differs from *C.himalaica* in awn (2.8–5.1 mm long, straight and filiform vs. 4.5–10 mm long, strongly geniculate) and from *C.nyingchiensis* in anther length (1.5–2.3 mm long vs. 0.7–1.1 mm long). *Calamagrostisnagarum* differs from *C.elatior* by having callus hairs shorter than half of the length of lemma and, straight and filiform awn.

### ﻿Taxonomic key to the *C.lahulensis*, *C.nagarum* and *C.scabrescens*

**Table:** 

1	Leaf blades pilose on adaxial surface; panicle branches widely spreading; nerve prolongation of lemma conspicuous with (0.3–)0.4–0.7(–0.9) mm long	***C.nagarum***
–	Leaf blades without hairs on adaxial surface; panicle branched ascending; nerve prolongation of lemma usually absents, if present then < 0.4 mm long	**2**
2	Leaf blades with prominent grooves and deep furrows; panicle (6–)11–18(–25) cm long; awns usually strongly geniculate, rarely straight with (3.3–)5.0–6.8(–7.8) mm long	***C.scabrescens***
–	Leaf blades without prominent grooves and deep furrows: panicles (1.5–)4–7(–9.0) cm long; awns not geniculate and straight with (1.6–)2.3–3.2(–4.5) mm long	***C.lahulensis***

### ﻿Typification of names

#### ﻿1. Calamagrostis lahulensis

Taxon classificationPlantaePoalesPoaceae

G.Singh, in Taxon 33 (1) 94 (1984)

BAE100AA-8AA1-5996-B384-40BE03623890

##### Replaced name

**(typonym).***Calamagrostispulchella* Griseb. Nachr. Ges. Wiss. Göttingen, Math. -Phys. Kl., 78 (1868), non Saut. ex Rchb. Fl. Germ. Excurs.: 26 (1830)

##### Lectotype

**(designated here).**–India. Sikkim, Guantong, 12,000 ft. [3657 m], 5 September 1849, [*Deyeuxia* 10], *J.D. Hooker* s.n. (K000032374; digital image!). (Image available at https://plants.jstor.org/stable/10.5555/al.ap.specimen.k000032374)

##### Note.

*Calamagrostislahulensis* was originally described by Grisebach (1868) as *C.pulchella*. Later, Hooker (1896) assigned *C.pulchella* to the genus *Deyeuxia*. Thereafter, Singh (1984) transferred the Indian species of *Deyeuxia* into *Calamagrostis* and proposed a new name *C.lahulensis* for *C.pulchella* Griseb. as the epithet “*pulchella*” was preoccupied in *Calamagrostis* by the name *C.pulchella* Saut. ex Rchb. Grisebach (1868) proposed the name *C.pulchella* based on the heterogenous collections of Hooker, covering the regions between Garhwal and Sikkim Himalaya of India. Although all specimens seen by Grisebach, were ‘sino numero’ (without collection number), he cited localities, altitude, date of collection, name of the collector, and annotation “10 *Deyeuxia*” in the protologue. While searching for the original specimens in various herbaria (BM, CAL, DD, E, K and P.), where most of the Hooker’s specimens are housed, we traced seven specimens at K (K000032374, K000032375, K000032376, K000032377, K000838345, K0000838346, and K0000838348), three specimens at CAL (CAL0000002397, CAL0000002398, and CAL0000002399), four specimens at W (W0026815, W1889-0241774, W1889-0038498, and W1916-00037771) and one specimen at E (E00394124). All the specimens have a label consisting of the collection details with the annotation ‘10 *Deyeuxia*’and matched well with the protologue. Therefore, they should have been considered as syntypes (Art. 9.6 of the ICN; Turland et al. 2018). The specimens housed at K have been verified by H.J. Noltie as syntypes, while the specimens preserved at W and CAL have been verified by Beata Paszko, Polish Academy of Sciences Poland (KRAM) as type materials. In addition to these, L. Pignotti has also identified the specimens housed at W as syntypes. Another specimen housed at K (K000838347) was from East Nepal, and thus we have excluded it from type materials. We have also examined the type specimens of *C.lahulensis* housed at CAL, which has the same morphological characters, such as panicle short with about 3–4 cm long and congested and awn straight and shortly or not exserted from the spikelet as found in the typical form of *C.lahulensis*. Since Grisebach (1868) examined the grass specimens which were housed at Kew herbarium for the description of *C.lahulensis*, the nomenclatural type should be from the Kew specimens. The presence of rhizomes, culms, ligules, inflorescences, and spikelets and its good preservation makes the specimen K000032374 suitable to choose as the lectotype; the same has been designated here lectotype for the name *C.lahulensis* following Art. 9.3 of the ICN (Turland et al. 2018).

#### ﻿2. Calamagrostis scabrescens

Taxon classificationPlantaePoalesPoaceae

Griseb., Nachr. Königl. Ges. Wiss. Georg-Augusts-Univ. 3: 79. (1868)

96EA7DED-6744-5D96-BA76-9826A89C5E42

C. scabrescens var. humilis Griseb. Nachr. Königl. Ges. Wiss. Georg-Augusts-Univ. 3: 79 (1868). Lectotype (designated here):–India. Sikkim, Lachen, 11,000 ft. [3352.8 m], [D. nr. 9], 3 July 1849, J.D. Hooker s.n. (K: K000838368, digital image!). (Image available at https://plants.jstor.org/stable/viewer/10.5555/al.ap.specimen.k000032368).

##### Lectotype

**(designated here).**–India. Sikkim, Lachen, 12,000 ft [3600 m], [*Deyeuxiascabrescens* Munr.], 3 August 1849, *J.D. Hooker s.n.* (K: K000838333, digital image!). (Image available at https://plants.jstor.org/stable/viewer/10.5555/al.ap.specimen.k000838333)

##### Note.

Grisebach (1868) described *Calamagrostisscabrescens* with three varieties viz. (α) var. scabrescens Griseb., (β) var. elatior Griseb. and (γ) var. humilis Griseb. based on Hooker’s gatherings which were housed in Kew Herbarium. The name *C.scabrescens* (≡var. scabrescens) was given for H. [Hooker’s]: *D.* [*Deyeuxia*] *scabrescens* Munr., characterized by ciliated glumes along the margin. The var. elatior was proposed for H. [Hooker’s]: *D.* [*Deyeuxia*] nr. 7, in which glumes are not ciliated along the margin, panicle sub-violet and about 30 cm long. Var. humilis was proposed for the H. [Hooker’s]: D. [*Deyeuxia*] nr. 9, which differs from the above by having non ciliated glumes, panicle narrow and greenish, and ligule short, truncate or obtuse. Later, var. humilis was synonymized with *C.scabrescens*, while var. elatior raised to rank of species as *C.elatior* (Griseb.) A. Camus (Camus 1928; Bor 1960).

While searching for Hooker’s specimen(s) belonging to the name *C.scabrescens* in various herbaria we traced six specimens, two at K (K000838333 and K000838334), one specimen at BM (BM000573477), CAL (0000004002), MPU (MPU027066), and W (W1889-0241775) each. All the specimens were collected from the different localities of Sikkim Himalaya and have the annotation “H.: *Deyeuxiascabrescens* Munr.” The specimens at K, W, and CAL were verified as type materials by Beata Paszko. We have examined all the specimens including those which are housed in MPU and BM and determined as type material of *C.scabrescens*.

At CAL, the specimen, CAL0000002402, was stored under the type materials of *C.scabrescens*, without annotation of “*Deyeuxiascabrescens* Munr.” on the sheet. This specimen was identified as the type of *Deyeuxiafiliformis* senso. Hook. f., but later identified as *C.scabrescens* by Sunanda Bhattacharya, Botanical Survey of India. According to Paszko (2012a), it belongs to *Deyeuxiafiliformis* senso. Hook. f. of *C.lahulensis*-*C.scabrescens* complex. Therefore, we excluded it from the type materials.

Since Grisebach examined Hooker’s specimens housed at K for his new species *C.scabrescens*, the specimen with barcode K000838333 (left-hand side) is designated here as lectotype for the name *C.scabrescens* as per Art. 9.3 of the ICN (Turland et al. 2018), because of its good preservation with complete plants including inflorescence and spikelet and illustration of ciliated glumes on the sheet.

In addition to this, for the var. humilis we have traced another two specimens at K (K000838352 and K000032368) and one specimen at W (W0026817) which belong to the type materials of var. humilis. All of them bear the annotation “H.: D. nr. 9”, and thus should be considered as syntypes (Art. 9.6 of the ICN; Turland et al. 2018). The specimen with barcode K000838368 is designated here as the lectotype for the name C.scabrescensvar.humilis following Art. 9.3 of the ICN (Turland et al. 2018), as the specimen is well-preserved and also morphologically complete with roots, inflorescences, and spikelets.

## Supplementary Material

XML Treatment for Calamagrostis nagarum

XML Treatment for Calamagrostis lahulensis

XML Treatment for Calamagrostis scabrescens

## ﻿Appendix 1

***C.lahulensis***: INDIA. **Himachal Pradesh**, Kullu, Manali, near bridge of Marhi, 32.348869N, 77.223234E, 3372 m, 7 August 2019, *D Prasad, R Yadav & P Rajput 326811* (LWG!); near bridge of Marhi, 32.348869N, 77.223234E, 3390 m, *D Prasad, R Yadav & P Rajput 326811* (LWG! ?); 10 km before from Rohtang Pass, 32.35789N, 77.21695E, 3635 m, 5 August 2019, *D Prasad, R Yadav & P Rajput 316253, 316250* (LWG!); Marhi, 32.341507N, 77.216715E, 3260 m, 5 August 2019, *D Prasad, R Yadav & P Rajput 316299* (LWG!); Manali, 1 km after Gulaba check post, on the way to Marhi, 32.319782N, 77.20379E, 2944 m, *D Prasad, R Yadav & P Rajput 326868* (LWG!); **Uttarakhand**, Chamoli, Valley of Flowers National Park, 30.711627N, 79.595142E, 3250 m, 23 August 2019, *D Prasad, R Yadav, S Jaiswal & P Agnihotri* 326641 (LWG!); Valley of Flowers National Park, 30.711627N, 79.595142E, 3380 m, 23 August 2019, *D Prasad, R Yadav, S Jaiswal & P Agnihotri 326642* (LWG!); Valley of Flowers National Park, 2 km after Ghanghria, 30.7114N, 79.5962E, 3137 m, 23 August 2019, *D Prasad, S Jaiswal, R Yadav & P Agnihotri 326698* (LWG!); Valley of Flowers National Park, 30.711627N, 79.595142E, 3380 m, 23 August 2019, *D Prasad, S Jaiswal, R Yadav & P Agnihotri 326642* (LWG!); Valley of Flowers of National Park, 30.729139N, 79.596606E, 3567 m, 23 August 2019, *D Prasad, R Yadav, S Jaiswal & P Agnihotri 326628* (LWG!); **Sikkim**, Gangtok, 13000ft [3962.4 m], [9 *Deyeuxia*], 6 September 1849, *JD Hooker s.n.* (K, K000838352); no specific locality, 15000ft [4572 m], 12 August 1913, *GH Gave 975* (CAL!); no specific locality, 14500 ft [4419.6 m], 7 October 1869, [Trisetum?], *sc.l. s.n.* (CAL!); no specific locality, 13000ft [3962.4 m], September 1885, *G King* s.n. (CAL!); no specific locality, 16000ft [4876.8 m], 19 August 1892, *GA Gammie s.n.* (CAL!); **Meghalaya**, Shillong, Kamegh, Sangetsar lake road, on hill slope, 19 August 1976, *PK Hajra 68327* (CAL!) (Seen rosette spikelet); Pankensaw, Nahula road, 16 August 1976, *PK Hajra 68551* (CAL!). **Nagaland**, Khasia, Khasi Hills, 13 September 1883, *CB Clarke 40446* (CAL!); **NEPAL.** no specific locality, alpine habitat, 12–16000ft [3657.6-4876.8 m], [10 *Deyeuxia*], *JD Hooker s.n.*, (K, K000838347); East Nepal, Chaika Pahar, 13000ft [3962.4 m], 25 September 1954, *Stainton, Sykes & Williams 4583* (CAL!); Chaika Pahar, 15000ft [4572 m], 22 September 1954, *Stainton, Sykes & Williams 4546* (CAL!); Gyang, Kyangsin, 13,500ft [4114.8 m], August 1949, *O. Polunin 1682* (CAL!); Sauwla Khola, 12,500ft [3810 m], 23 July 1954, *Stainton, Sykes & Williams 3606* (CAL!); **BHUTAN.** GaFoola, upper Phu Chu, 14500ft [4419.6], July 1949, *F Ludlow, G Sheriff & JH Hicks 16761* (CAL!); without specific locality, 12000ft [3657.6 m], *sc.l. s.n.*, (CAL! ac. no. 56614), (intermix with *C.scabrescens*).

***C.nagarum***: INDIA. Uttarakhand, Bageshwar, Pindari Valley, Dwali, 30.1783N, 79.9958E, 2800 m, 28 September 2021, *D Prasad, S Sharma, K Yadav & P Dey 339324* (LWG!): same locality, 28 September 2021, *D Prasad, S Sharma, K Yadav & P Dey 339325* (LWG!); Phurkia, 30.2192N, 80.0002E, 3300 m, 30 September 2021, *D Prasad, S Sharma, K Yadav & P Dey 339372* (LWG!); same locality, 30 September 2021, *D Prasad, S Sharma, K Yadav & P Dey 339374* (LWG); Dhakuri top, 30.072219N, 79.920847E, 2900 m, 21 August 2022, *Ravindra Kumar 342005* (LWG!).

***C.scabrescens***: INDIA. **Jammu & Kashmir**, Hazara, 26 July 1899, *Inaiyat s.n.* (CAL!); **Himachal Pradesh**, Kullu, Manali, 12 km after Marhi, on the way to Rohtang pass, 32.356838N, 77.222554E, 3013 m, 7 August 2019, *D Prasad, R Yadav & P Rajput 326829*, (LWG); 12 km after Marhi, on the way to Rohtang Pass, 32.356858N, 77.222554E, 7 August 2019, *D Prasad, R Yadav & P Rajput 326842* (LWG); Marhi, on the way to Rohtang pass, 32.3568N, 77.2225E, 3528 m, 7 August 2019, *D Prasad, R Yadav & P Rajput 326843* (LWG!); Marhi, 32.34150N, 77.216715E, 3260 m, 5 August 2019, *D Prasad, R Yadav & P Rajput 326888* (LWG!); on the way to Marhi, 32.3565N, 77.2225E, 3528 m, 7 August 2019, *D Prasad, R Yadav & P Rajput 314813* (LWG); Kinnaur, Nachar, upper Bahshar, 10,000ft [3048 m], 25 September 1858, *PC Nanda 1802* (CAL!); **Uttarakhand**, Garhwal, Sri Nagar, University Campus, 30.226388N, 78.5022E, 587 m, 22 September 2018, *S Tripathi 315803*, (LWG!); Chamoli, Valley of Flowers National Park, 30.711895N, 79.595247E, 3428 m, 23 August 2019, *D Prasad, R Yadav, S Jaiswal & P Agnihotri 326657*, (LWG!); Valley of Flowers National Park, 30.711077N, 79.596004E, 3417 m, 23 August 2019, *D Prasad, R Yadav, S Jaiswal & P Agnihotri 326657*, (LWG!); Chamoli, Ghanghria, on the way to Himkund, 30.70594N, 79.598963E, 3224 m, 22 August 2019, *D Prasad, R Yadav, S Jaiswal & P Agnihotri 326717*, (LWG!); Valley of Flowers National Park, 30.711896N, 79.59524E, 3296 m, 23 August 2019, *D Prasad, R Yadav, S Jaiswal & P Agnihotri 326651* (LWG!); Ghanghria, on the way to Himkund, 30.705944N, 79.59896E, 3224 m, 22 August 2019, *D Prasad, R Yadav, S Jaiswal & P Agnihotri 326729* (LWG!); Valley of Flowers National Park, 30.7059N, 79.6022E, 3438 m, 23 August 2019, *D Prasad, R Yadav, S Jaiswal & P Agnihotri 326683*, (LWG!); Valley of Flowers of National Park, 30.712096N, 79.592776E, 3417 m, 23 August 2019, *D Prasad, R Yadav, S Jaiswal & P Agnihotri 326863* (LWG!); 2 km after Ghanghria, on the way to Valley of Flowers, 30.71114N, 79.596208E, 3137 m, 23 August 2019, *D Prasad, R Yadav, S Jaiswal & P Agnihotri 326691* (LWG!); Valley of Flowers National Park, 30.712096N, 79.592776E, 3417 m, 23 August 2019, *D Prasad, R Yadav, S Jaiswal & P Agnihotri 326745* (LWG!); Pithoragarh, Kali Valley, 12000ft [3657.6 m], 15 September 1884, *JF Duthie 3584* (CAL!); Kumaon, Bageshwar, Dwali, 10-11000ft [3048-3352.8 m], 6 August 1886, *JF Duthie 6216* (CAL); Pithoragarh, Malpa, 2200-2500 m, 12 June 1960, *TA Rao 1178* (CAL!); Tehri-Garhwal, Rhudaghara, 10-11000ft [3048- 3352.8 m], *JF Duthie 145* (CAL!); Garhwal, Jaunsar, 8500ft [2590.8 m], September 1898, *JS Gamble 27238* (CAL!); Kumaon, 4,300 m, 10 August 1972, *CM Arora 49828* (LWG!); Pithoragarh, Kali Valley, 12-13000ft [3657.6-3962.4 m], 27 July 1888, *JF Duthie 6223* (CAL!); Kumaon, 14-15000ft [4267.2-4572 m], 31 August 1884, *JF Duthie 3538* (CAL!); Sansal-Nala, Killar Valley, 13-14000ft [3962.4-4267.2 m], 31 July 1893, *JF Duthie13349* (CAL!); Teyum, Haya, 4300 m, 10 August 1972, *CM Arora 49826*, (CAL!); BhojPass, 3600 m, 8 September 1972, *S. cl, 69* (CAL!); **Sikkim**, Kupup-Chango road, 13300ft [4114.8 m], 10 October 1928, *NL Bor 487* (CAL!);without precise locality, 12000ft [3657.6 m], 27 July 1910, *WW Smith 3875* (CAL!); Gangtong, 13000ft [3962.4 m], 21 September 1926, *NL Bor 153* (CAL!); **West Bengal**, Darjeeling, 11917ft [3632.3 m], 27 June 1960, *AB Chaoudhary 33* (CAL!); Darjeeling, 12000ft [3657.6 m], *JS Gamble s.n*, (CAL!); Meghalaya, Shillong, Kamegh, Sangetswar-ZImithang road, 20 August 1976, *PK Hajra 68351* (CAL!). **North-East**, 3600 m, 19 September 1962, *B. Safui 1781*, (CAL!); BHUTAN, without precise locality, *Griffith 6599* (CAL!); NEPAL. Muktinath, on open slope, 12,500ft [3810 m], 26 July 1954, *Stainton, Sykes & Williams 1429* (CAL!); Near Tarakot, 10,000 m [3048 m], 10 July 1990, *O Polunin, WR Sykes & LHJ Williams 2426* (CAL!); North of Muktinath, Damoda Kund, 14,000ft [4267.2 m], 30 July 1954, *Stainton, Sykes & William 2102, 7371* (CAL!).

## Supplementary materials

Supplementary material 1 Quantitative data of morphological characters and elevation data of *Calamagrostislahulensis*, *C.nagarum*, and *C.scabrescens* Data type table Explanation note Morphological and Elevation (m). https://binary.pensoft.net/file/765897This dataset is made available under the Open Database License (http://opendatacommons.org/licenses/odbl/1.0/). The Open Database License (ODbL) is a license agreement intended to allow users to freely share, modify, and use this Dataset while maintaining this same freedom for others, provided that the original source and author(s) are credited.Author: Dileshwar Prasad
